# Supplementary material for: A two-step activation mechanism enables mast cells to differentiate their response between extracellular and invasive enterobacterial infection
Source: Nat Commun. 2024 Jan 30;15:904. doi: 10.1038/s41467-024-45057-w (PMC10828507; doi:10.1038/s41467-024-45057-w)
Supplement: Supplementary file 1 — Supplementary Information [file 41467_2024_45057_MOESM1_ESM.pdf]

Supplementary information for:

A Two-Step Activation Mechanism Enables Mast Cells to Differentiate their Response between Extracellular and Invasive Enterobacterial Infection

Christopher von Beek[1], Anna Fahlgren[2], Petra Geiser[1], Maria Letizia Di Martino[1], Otto Lindahl[1], Grisna I. Prensa[1], Erika Mendez-Enriquez[1], Jens Eriksson[1], Jenny Hallgren[1], Maria Fällman[2], Gunnar Pejler[1]*, Mikael E. Sellin[1,3]*

Affiliations

[1]Department of Medical Biochemistry and Microbiology, Uppsala University, Uppsala, Sweden.

[2]Department of Molecular Biology, Laboratory for Molecular Infection Medicine Sweden (MIMS), Umeå Centre for Microbial Research (UCMR), Umeå University, Umeå, Sweden.

[3]Science for Life Laboratory, Uppsala, Sweden.

Correspondence: G.P. gunnar.pejler@imbim.uu.se; M.E.S. mikael.sellin@imbim.uu.se

Supplementary Figure 1

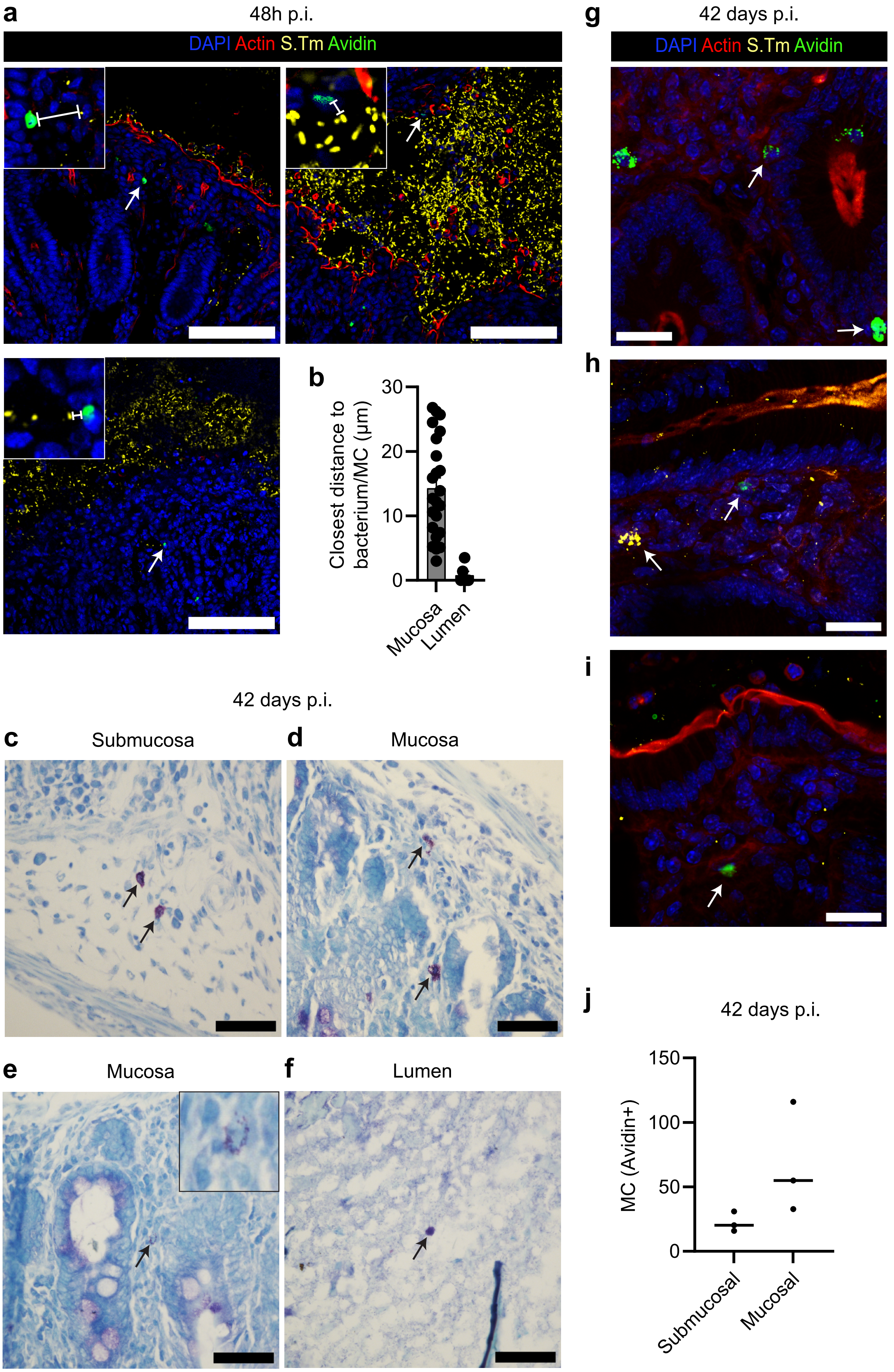

Supplementary Figure 1. Mast cells can be found in close proximity to *S.*Tm in the infected gut mucosa. A: Representative IF images of *S*.Tm^{wt} 1344-infected caecum tissue 48h p.i., used in quantification of the distances of individual MCs to their closest bacterium. Scale bars: 100µm. Magnifications are in 50x50µm for the top left image and 25x25µm for the other two images. Arrows indicate magnified MCs. **B**: Quantification of distances to closest bacteria for individual MCs within fields of view containing both MCs and *S*.Tm. For individual MCs (23 in mucosa and 6 in the lumen), a straight line was drawn to the closest bacterium and the distance measured. Bars show mean ± SEM. **C**-**F**: Representative images of toluidine blue-stained tissue sections of caecum from mice 42 days p.i. with *S*.Tm^{wt} SL1344. Arrows indicate different MC locations, such as submucosa (C), mucosa (D-E) and lumen (F). Scale bars: 50µm. **G**-**I**: Representative maximum intensity projection IF images of caecum tissue from mice 42 days p.i.. Arrows indicate the position of MCs or, in case of H, a pocket of *S*.Tm. Scale bars: 25µm. **J**: Quantification of avidin+ cells per caecum section at 42 days p.i., indicated as submucosal and mucosal MCs. Every dot indicates the mean of at least 6 sections for one mouse, n = 3. Horizontal lines display median.

Supplementary Figure 2

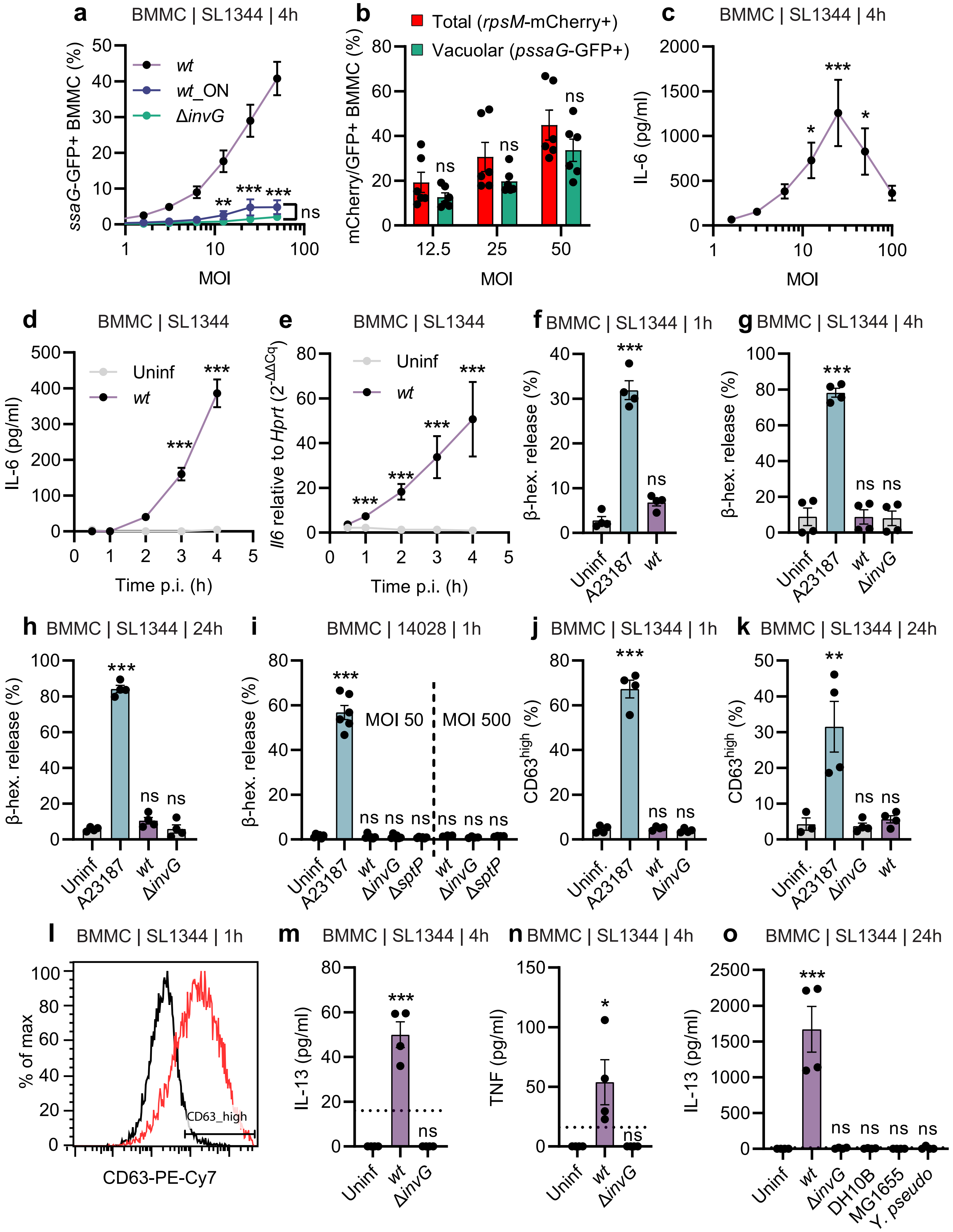

Supplementary Figure 2. Mast cells respond to invasive *Salmonella* infection by cytokine gene transcription and secretion, but negligible degranulation. A: MOI-dependent quantification of BMMCs harboring vacuolar *S.*Tm. "ON" indicates that the *S.*Tm inoculum was grown as an over-night stationary phase culture **B**: Quantification of BMMCs bound to or invaded by *S.*Tm (red bars) compared to BMMCs harboring vacuolar *S.*Tm (green bars). **C**: MOI-dependent quantification of IL-6 secretion by BMMCs, infected with *S.*Tm ^{wt} SL1344 for 4h. **D-E**: IL-6 secretion (**D**) and *Il6* transcript levels (**E**) of BMMCs left uninfected or infected with MOI 50 of *S.*Tm^{wt} SL1344 for the indicated time frames. **F-H**: β-hexosaminidase release from BMMCs as an indicator of degranulation 1h (F), 4h (G) or 24h (H) p.i. with MOI 50 of the indicated *S.*Tm SL1344 strains. A23187 served as positive control. **I**: Similar setup as in F, but with *S.*Tm^{wt} or the indicated TTSS-mutants of strain 14028, at MOI 50 and MOI 500. **J**-**K**: CD63^{high} cell percentage as indicator of degranulated BMMCs 1h (J) and 24h (K) after infection with MOI 50 of the indicated *S.*Tm SL1344strains. A23187 served as positive control. **L**: Representative gating for J-K. Black: untreated BMMCs, red: 1h A23187. **M-N**: IL-13 (M) and TNF (N) secretion from BMMCs, 4h after infection with MOI 50 of S.Tm^{wt} SL1344 or the indicated TTSS-mutants. **O**: Secreted IL-13 from BMMCs infected with MOI 50 of S.Tm^{wt} and *S.*Tm$^{\Delta invG}$ SL1344 as well as *E. coli* DH10B, *E. coli* MG1655 and *Y. pseudotuberculosis* for 24h. Every experiment was performed 2-3 times and mean ± SEM of pooled biological replicates is shown. Groups in A were statistically analyzed with two-way ANOVA and Tukey's posthoc test (every group for each MOI to "*wt*" group). Groups in B were analyzed with two-way ANOVA and Dunnet's posthoc test (total to vacuolar group). For C, F-K, M-O; one-way ANOVA together with Dunnet's posthoc test was used with uninfected cells being compared to all other groups. For D-E, two-way ANOVA with Sidak's posthoc test was used to compare uninfected cells with *S.*Tm^{wt}-infected cells within each time point. * $p<0.05$; ** $p<0.01$; *** $p<0.001$; ns – non-significant. Exact n- and p-values can be derived from the source data. For ATCC 14028 infections, a Δ*malX* strain was used as *wt*.

Supplementary Figure 3

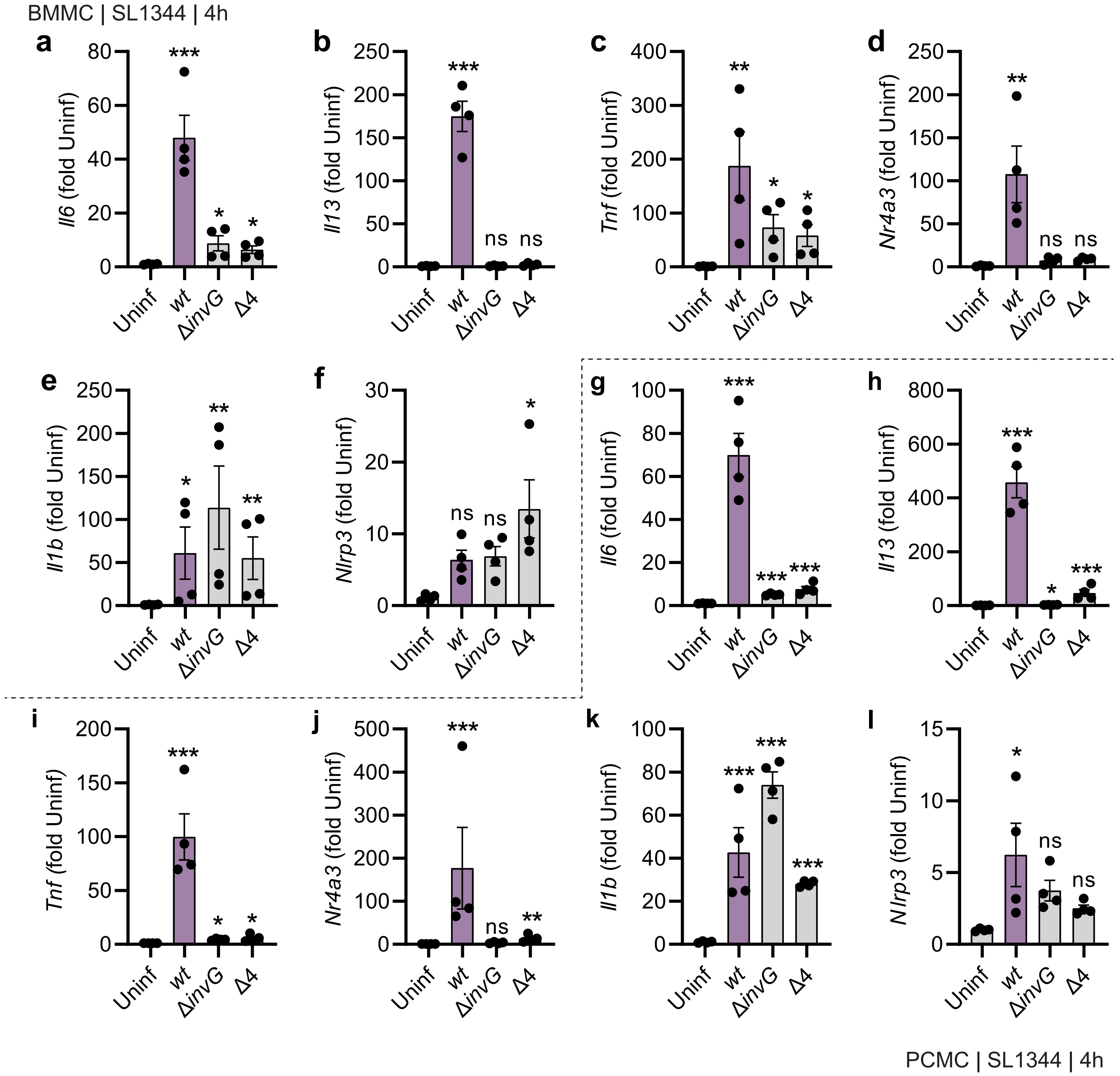

Supplementary Figure 3. Distinct transcriptional profiles of mast cells infected with invasive vs. non-invasive *Salmonella*. A-F RT-qPCR quantification of transcript levels in BMMCs, 4h after infection with MOI 50 of *S.*Tm^{wt} SL1344 or the indicated TTSS-mutants. **G-L:** Similar as in A-F, but PCMCs were used. Every experiment was performed 2-3 times and mean ± SEM of pooled biological replicates is shown. Data was statistically analyzed with one-way ANOVA and Dunnet's posthoc test, using uninfected cells for comparisons to all other groups. * p<0.05; ** p<0.01; *** p<0.001; ns – non-significant. Exact n- and p-values can be derived from the source data. A selection of these data (A-D, G-J) are also summarized in heatmaps in Figure 2H-I.

Supplementary Figure 4

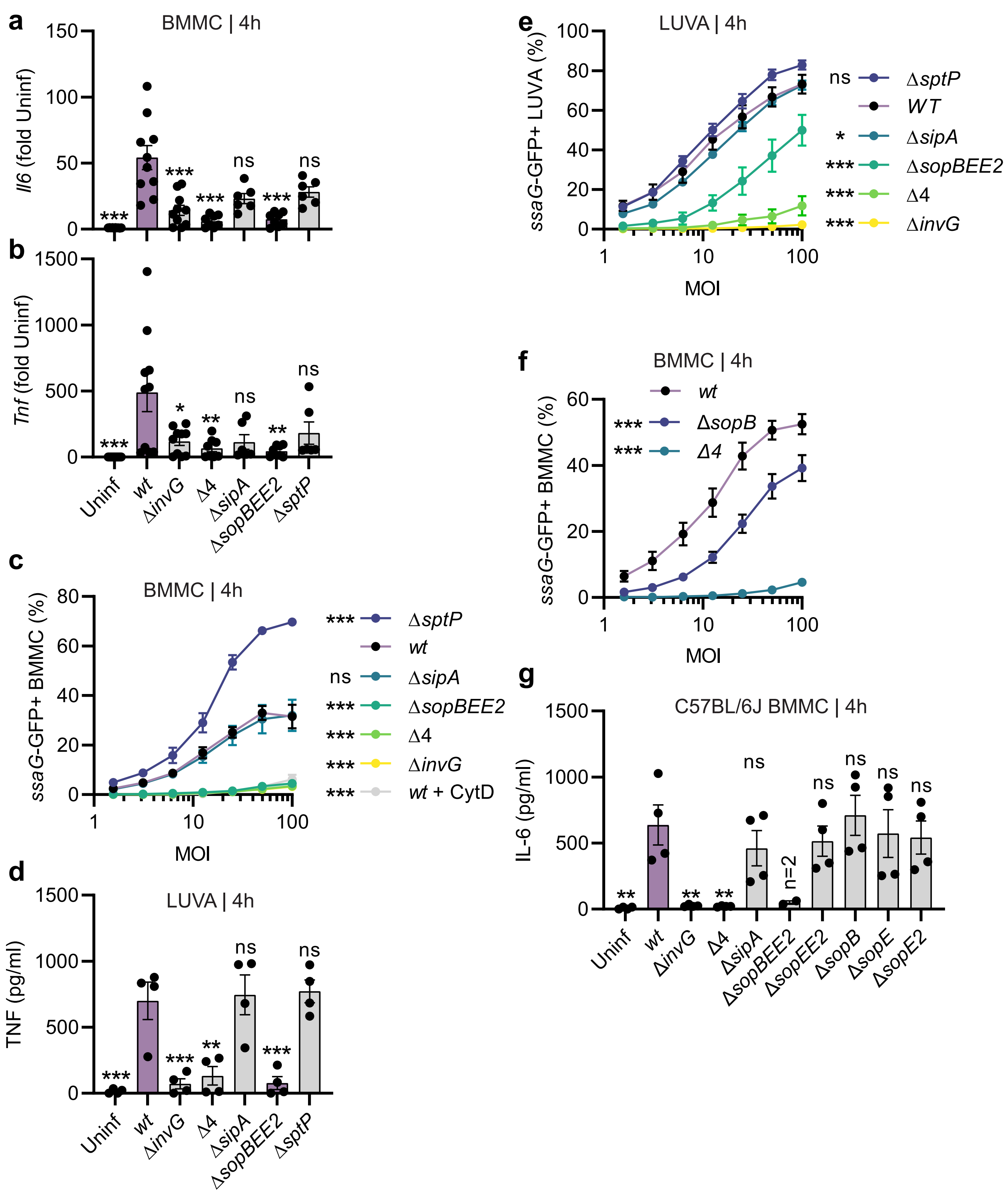

Supplementary Figure 4. The TTSS-1 effectors SopB, SopE, and SopE2 promote murine and human mast cell cytokine secretion upon *Salmonella* infection. A-B: RT-qPCR quantification of *Il6* (**A**) and *Tnf* (**B**) transcript levels in BMMCs infected with MOI 50 of *S*.Tm^{wt} SL1344 or the indicated TTSS-mutants for 4h. **C, F**: Quantification of the MOI-dependent frequency of BMMCs harboring vacuolar bacteria, 4h after infection with MOI 50 of S.Tm^{wt} SL1344 or the indicated TTSS-mutants. For actin inhibition, BMMCs were pretreated with 1µM of Cytochalasin D for 1h. **D**: TNF secretion from LUVA cells, infected with MOI 50 of S.Tm^{wt} SL1344 or the indicated TTSS-mutants for 4h. **E**: Quantification of the MOI-dependent frequency of LUVA cells harboring vacuolar bacteria, 4h after infection with MOI 50 of S.Tm^{wt} SL1344 or the indicated TTSS-mutants **G**: IL-6 secretion from C57BL/6J (Jackson) BMMCs infected with MOI 50 of *S*.Tm^{wt} SL1344 or the indicated TTSS-mutants for 4h. Every experiment was performed 2-3 times and mean ± SEM of pooled biological replicates is shown. For A, B, D and G, data was statistically analyzed with one-way ANOVA and the Dunnet's posthoc test, using *S*.Tm^{wt}-infected cells for comparison to all other groups. For C, E, and F, two-way ANOVA was used with Sidak's posthoc test to compare the respective mutants and *S*.Tm^{wt}. * p<0.05; ** p<0.01; *** p<0.001; ns – non-significant. Exact n- and p-values can be derived from the source data.

Supplementary Figure 5

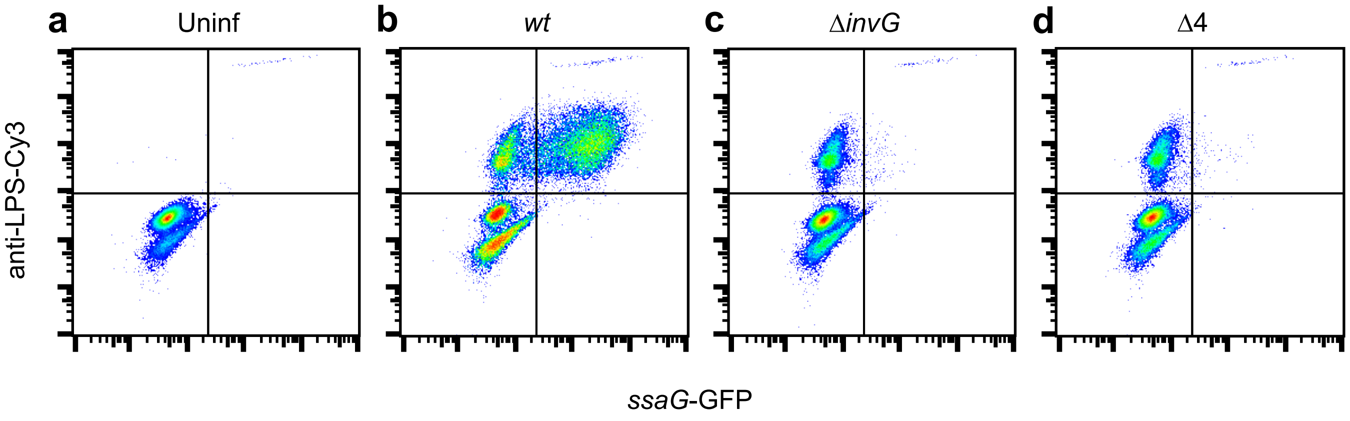

Supplementary Figure 5. Flow cytometry gating for data shown in Figure 4B. A-D: Representative flow cytometry gating for quantification of MCs positive for vacuolar *S*.Tm (*ssaG*-GFP+) and/or *S*.Tm LPS. BMMCs were infected with MOI 50 of *S*.Tm^{wt} SL1344 or the indicated TTSS-mutants for 4h prior to analysis by flow cytometry.

Supplementary Figure 6

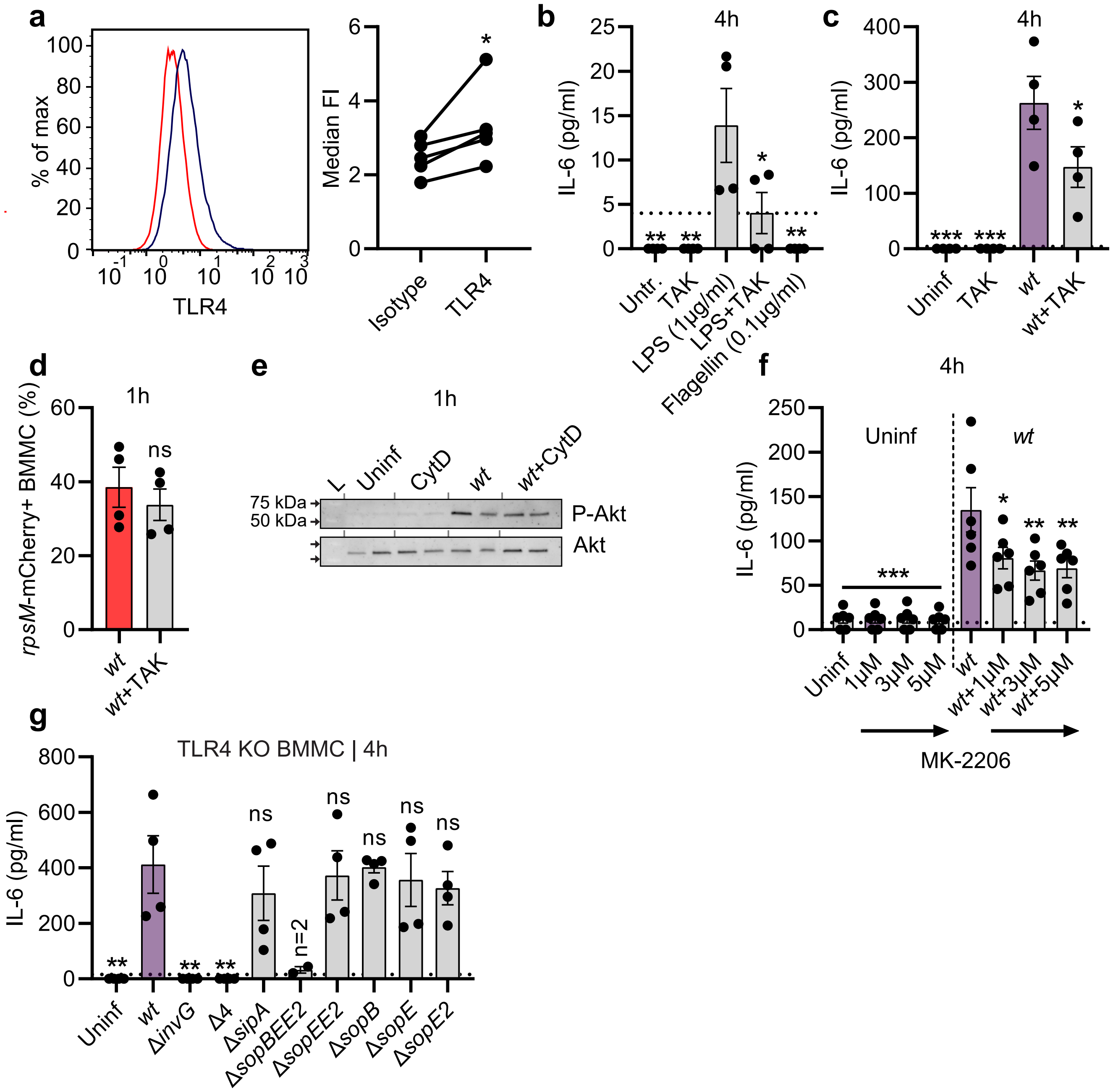

Supplementary Figure 6. Mast cells express surface TLR4 and react to *Salmonella* through pathways involving TLR4 and Akt. A: Representative surface detection of TLR4 (blue) and isotype control (red) in BMMCs. Pairwise comparisons shown in right panel. **B**: IL-6 secretion from BMMCs 4h after treatment with LPS or recombinant flagellin and/or pretreatment for 30-45min with TAK-242. **C**: IL-6 secretion from BMMCs 4h after infection with MOI 50 of *S*.Tm^{wt} SL1344 and/or pretreatment with TAK-242. **D**: Quantification of BMMCs harboring *S*.Tm 30min after infection. The indicated group was pretreated with TAK-242. **E**: P-Akt and Akt immunoblots of BMMCs after 1h infection with MOI 50 of *S*.Tm^{wt} SL1344 and/or pretreatment with 200nM Cytochalasin D. L = ladder. **F**: IL-6 secretion from BMMCs after 4h infection with MOI 50 of *S*.Tm^{wt} SL1344 and/or pretreatment with MK-2206 for 30-45min. **G**: IL-6 secretion from *Tlr4*$^{-/-}$BMMCs infected with MOI 50 of S.Tm^{wt} SL1344 or the indicated TTSS-mutants for 4h. Every experiment was performed 2-5 times and mean ± SEM of pooled biological replicates is shown. E shows the results from 1 immunoblot. For A (paired) and D (unpaired) t-tests were used (both two-sided). For B, C, F and G, data was statistically analyzed with one-way ANOVA and the Dunnet's posthoc test, using *S*.Tm^{wt}-infected cells (or LPS in case of B) for comparison to all other groups. * p<0.05; ** p<0.01; *** p<0.001; ns – non-significant. Exact n- and p-values can be derived from the source data.

Supplementary Figure 7

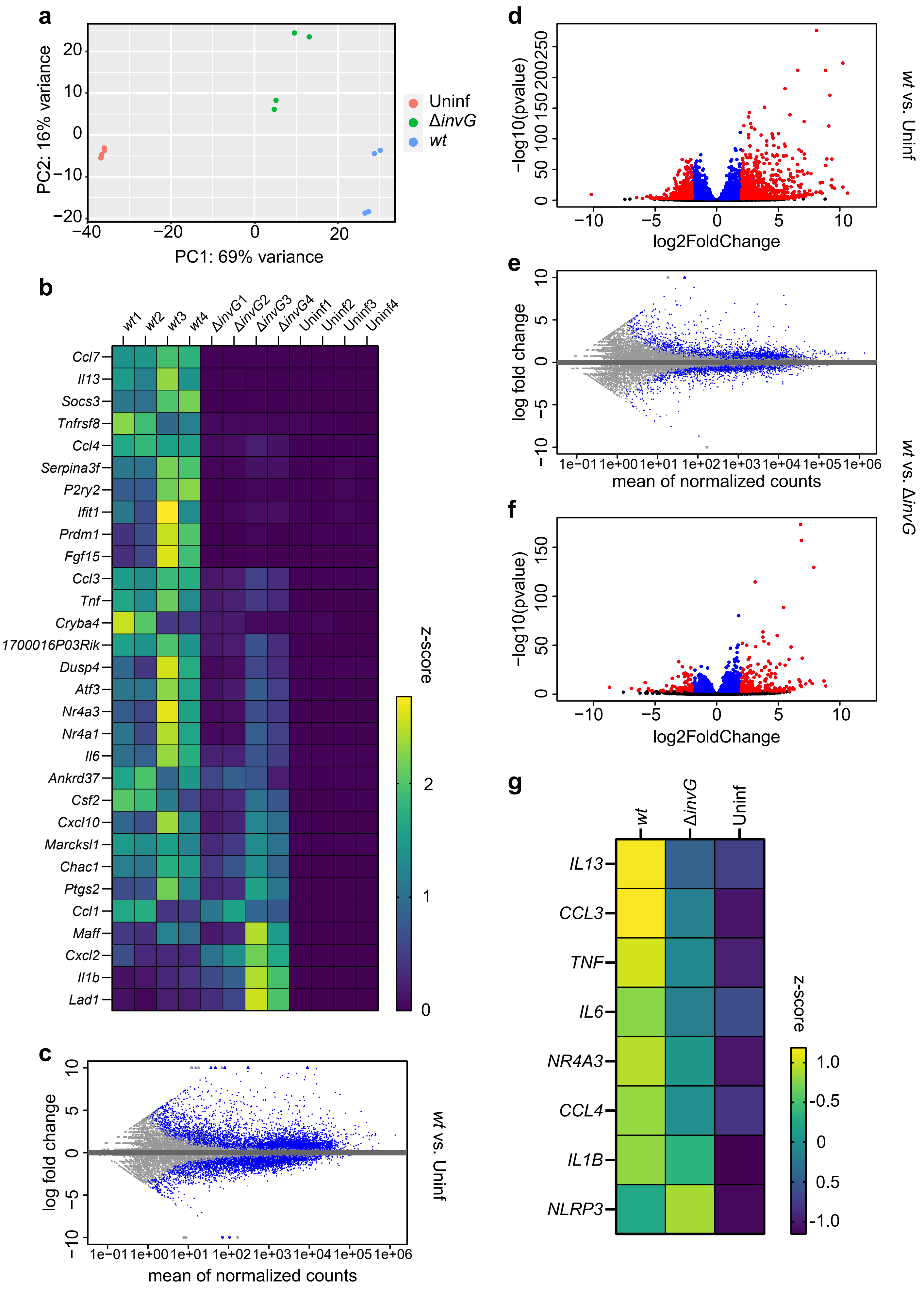

Supplementary Figure 7. Mast cells react to invasive vs. non-invasive *Salmonella* with distinct transcriptional responses. A: Principal component analysis plot of the RNA sequencing data described in Figure 6A. **B**: Complete heatmap corresponding to Figure 6A, but showing z-scores for all four biological replicates in each group. Replicates “1” and “2” stem from one experiment, while “3” and “4” stem from a separate experiment, performed on a different day. **C**-**F**: Bland–Altman (**C**, **E**) and volcano (**D**, **F**) plots for comparisons between *S*.Tm^{wt} and uninfected cells (**C**, **D**), or between *S*.Tm^{wt} and *S*.Tm$^{\Delta invG}$ (**E**, **F**). **G**: Mean transcript levels (RT-qPCR) of selected inflammatory mediators in human LUVA cells. Analogous to in B, LUVA cells left uninfected, or infected with MOI 50 of *S*.Tm^{wt} or *S*.Tm$^{\Delta invG}$ SL1344 for 4h, sorted by highest upregulated genes between *S*.Tm^{wt}-infected and *S*.Tm$^{\Delta invG}$ groups and displayed as a z-score-transformed heatmap. Experiments were performed two times with a total of 4 pooled biological replicates. Exact n-values can be derived from the source data.

Supplementary Figure 8

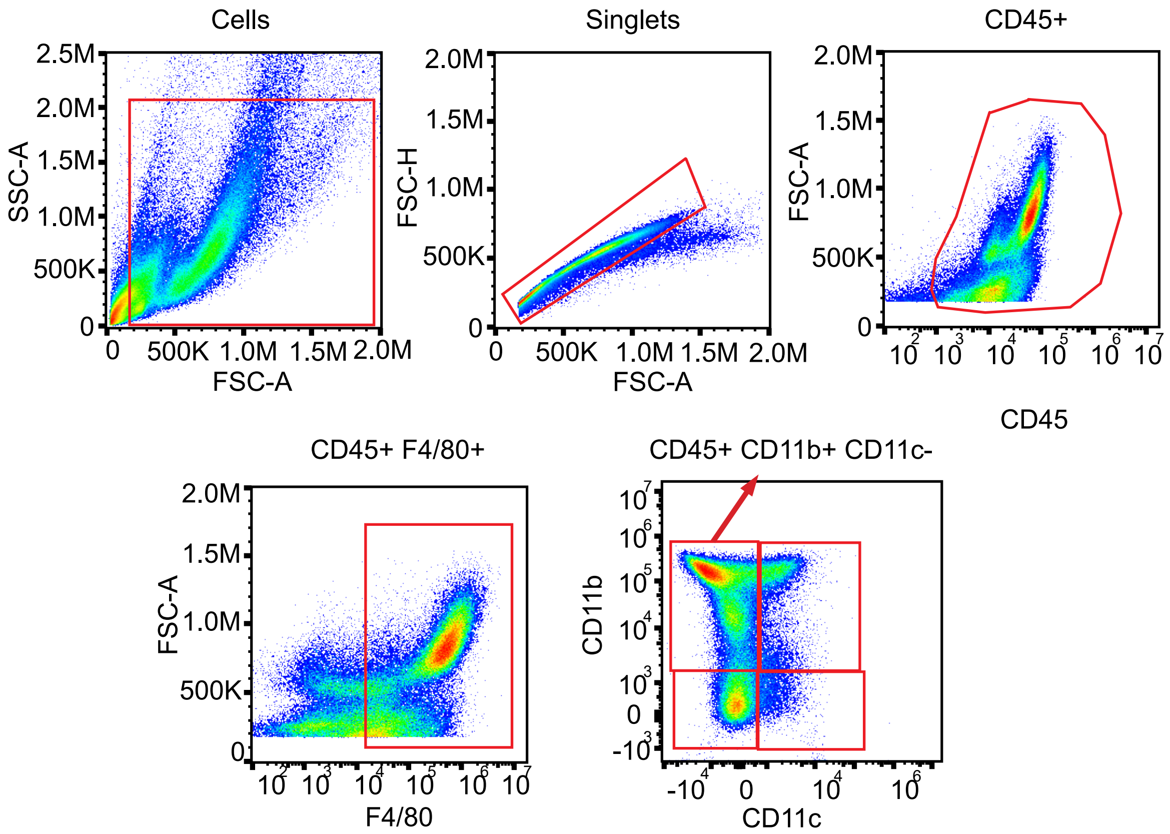

Supplementary Figure 8. Flow cytometry gating for data shown in Figure 6D-F. Representative flow cytometry gating strategy, depicting one of the bone marrow nucleated cell samples, cultured for 7 days in base medium supplemented with supernatant from BMMCs infected with *S.*Tm^{wt} for 24h.

Supplementary Table 1. Bacterial strains and mutants used in this study. Indicated resistances as “Sm” for streptomycin, “Cml” for chloramphenicol, “Km” for kanamycin, “Tet” for tetracycline, and “Nal” for nalidixic acid. Strains marked with “*” prior the genotype were also used with the p*ssaG*-GFP reporter plasmid (S2 Table). The pFPV-mCherry plasmid (S2 Table) was used together with SL1344 *S*.Tm^{wt}.

Strain	Genotype	Reference
S.Tm^{wt}	*SL1344, *wt* (SB300; Sm^{R})	1
S.Tm$^{\Delta invG}$	*SL1344, Δ*invG* (SB161; Sm^{R})	2
S.Tm$^{\Delta sipA}$	*SL1344, Δ*sipA* (M714; Sm^{R})	3
S.Tm$^{\Delta sopB}$	*SL1344, WITS17, Δ*sopB* (Sm^{R}, Km^{R}, Cml^{R}),	4
S.Tm$^{\Delta sopE}$	SB300 WITS2, Δ*sopE* (Sm^{R}, Cml^{R})	4
S.Tm$^{\Delta sopE2}$	SB300 WITS19, Δ*sopE2* (Sm^{R}, Km^{R}, Cml^{R})	4
S.Tm$^{\Delta sptP}$	*SB300 Δ*sptP* (Sm^{R}, Km^{R})	This study
S.Tm$^{\Delta sopEE2}$	SB300 WITS2, Δ*sopEE2* (Sm^{R}, Km^{R}, Cml^{R})	4
S.Tm$^{\Delta sopBEE2}$	*SL1344, Δ*sopB, sopE::aphT, sopE2::tet* (M516; Sm^{R}, Kan^{R}, Tet^{R})	5
S.Tm$^{\Delta sipA\ sopBEE2\ (\Delta 4)}$	*SL1344, Δ*sipA,* Δ*sopB,* Δ*sopE*, Δ*sopE2* (M566; Sm^{R}, Km^{R})	6
S.Tm$^{\text{"}wt\text{"}}$ 14028	14028, Δ*malX* (Kan^{R})	7
S.Tm$^{\Delta invG}$ 14028	14028, Δ*invG* (Kan^{R})	7
S.Tm$^{\Delta sipC}$ 14028	14028, Δ*sipC* (Kan^{R})	7
E. coli MG1655	Nal^{R}	8
E. coli DH10B	K12 DH10B (Sm^{R})	Thermo Fisher, #EC0113
Y. pseudotuberculosis	YPIII	9

Supplementary Table 2. Plasmids used in this study.

Plasmid	Reference
pFPV-mCherry	10
p*ssaG*-GFPmut2 high copy	11

Supplementary Table 3. Primers used for RT-qPCR in this study. Concentrations of primers was 200nM.

Species	Target	Name	Sequence	Efficiency (%)
Mouse	*Cpa3*	CPA3 F	GAA AGT TGC AAG GAT TGC CAC	99
Mouse	*Cpa3*	CPA3 R	TTG TGG ATG CTA TTG GGC CGT	99
Mouse	*Mcpt1*	mMcpt1 F	TCC TGA TGG CAC TTC TCT TGC	107
Mouse	*Mcpt1*	mMcpt1 R	TCC ACT ACA GTG TGC AGC AGT	107
Mouse	*Mcpt2*	mMcpt2 F	TGT GTG ATA GTG TGG CCC ATG	112
Mouse	*Mcpt2*	mMcpt2 R	TCT GAC TCA GGC TGG TTA GGC	112
Mouse	*Mcpt4*	mMcpt4 F	GCA GTC TTC ACC CGA ATC TC	83
Mouse	*Mcpt4*	mMcpt4 R	CAG GAT GGA CAC ATG CTT TG	83
Mouse	*Mcpt5*	mMcpt5 F	TCC CAC TCT CTG CCA ACT TCA	103
Mouse	*Mcpt5*	mMcpt5 R	TGG CTC ATT CAC GTT TGT TCT	103
Mouse	*Mcpt6*	mMcpt6 F	TGG CAT GCT GTG TGC TGG AAA	105
Mouse	*Mcpt6*	mMcpt6 R	AGG TAC CCT TCA CTT TGC AGA	105
Mouse	*Il6*	il6 F	AAGGGCTGCTTCCAAACCTTT	95
Mouse	*Il6*	il6 R	TGCCTGAAGCTCTTGTTGATG	95
Mouse	*Tnf*	tnf F	GAGCCCCCAGTCTGTATCCTT	102
Mouse	*Tnf*	tnf R	CCTGAGTTCTGCAAAGGGAGA	102
Mouse	*Nr4a3*	nr4a3 F	TCACCATCACCATCATCACCA	89
Mouse	*Nr4a3*	nr4a3 R	AAGGCGGAGACTGCTTGAAGT	89
Mouse	*Il1b*	il1b F	AAGGGCTGCTTCCAAACCTTT	85
Mouse	*Il1b*	il1b R	TGCCTGAAGCTCTTGTTGATG	85
Mouse	*Il13*	il13 F	AGG AGC TTA TTG AGG AGC TGA	95
Mouse	*Il13*	il13 R	TGG AGA TGT TGG TCA GGG AAT	95
Mouse	*Nlrp3*	nrlp3 F	ATTACCCGCCCGAGAAAGG	88
Mouse	*Nlrp3*	nlrp3 R	TCGCAGCAAAGATCCACACAG	88
Mouse	*Hprt*	hprt F	CCTAAGATGAGCGCAAGTTGAA	95
Mouse	*Hprt*	hprt R	CCACAGGACTAGAACACCTGCTAA	95

Supplementary Table 3. Primers used for RT-qPCR in this study (continued).

Species	Target	Name	Sequence	Efficiency (%)
Human	*GAPDH*	GAPDH F	GCATCTTCTTTTGCGTCG	103
Human	*GAPDH*	GAPDH R	TGTAAACCATGTAGTTGAGGT	103
Human	*TNF*	TNF F	CGCTCCCCAAGAAGAC	103
Human	*TNF*	TNF R	GGTTCGAGAAGATGATCTGA	103
Human	*IL1B*	IL1B F	TGATGGCCCTAAACAGATGAAG	116
Human	*IL1B*	IL1B R	ATCCAGAGGGCAGAGGTCC	116
Human	*NLRP3*	NLRP3 F	GATCTTCGCTGCGATCAACAG	105
Human	*NLRP3*	NLRP3 R	CGTGCATTATCTGAACCCCAC	105
Human	*IL6*	IL6 F	GTGAGTGGGATGGTGGAAGG	97
Human	*IL6*	IL6 R	CAAACTTGTGTGTTGCCCATTC	97
Human	*IL13*	IL13 F	CCTCATGGCGCTTTTGTTGAC	105
Human	*IL13*	IL13 R	TCTGGTTCTGGGTGATGTTGA	105
Human	*CCL4*	CCL4 F	CTGTGCTGATCCCAGTGAATC	104
Human	*CCL4*	CCL4 R	TCAGTTCAGTTCCAGGTCATACA	104
Human	*CCL3*	CCL3 F	AGTTCTCTGCATCACTTGCTG	113
Human	*CCL3*	CCL3 R	CGGCTTCGCTTGGTTAGGAA	113
Human	*NR4A3*	NR4A3 F	TGCGTCCAAGCCCAATATAGC	110
Human	*NR4A3*	NR4A3 R	GGTGTATTCCGAGCTGTATGTCT	110

Supplementary Table 4. Primary antibodies used in this study, with unique identifiers (RRID).

Antigen	RRID	Conjugate	Host	Catalogue no.	Clone	Provider	Dilution
Salmonella O Antiserum Factor 5	NA	NA	Rabbit	BD-226601	Polyclonal	Difco/MicLev	1:250
Akt	AB_329827	NA	Rabbit	9272	Polyclonal	Cell Signaling	1:1000
Phospho-Akt (Ser473)	AB_2315049	NA	Rabbit	4060	D9E	Cell Signaling	1:2000
CD16/32	AB_394657	NA	Rat	553142	2.4G2	BD Biosciences	1:1000
TLR4	AB_469944	Alexa Fluor 488	Mouse	53-9041-82	UT41	Invitrogen	1:100
Mouse IgG1 Isotype Control	NA	Alexa Fluor 488	Mouse	MG120	Polyclonal	Invitrogen	1:100
CD45	AB_1645208	Alexa Flour 700	Rat	560510	30-F11	BD Biosciences	1:100
CD11b	AB_468714	PE-Cy5	Rat	15-0112-82	M1/70	eBioscience	1:100
CD11c	AB_469590	PE-Cy7	Armenian hamster	25-0114-82	N418	eBioscience	1:100
F4/80	AB_465923	PE	Rat	12-4801-82	BMB	Invitrogen	1:100
CD45	AB_470499	NA	Rat	ab25386	I3/2.3	AbCam	1:50
CD18	AB_396701	NA	Rat	557437	M18/2	BD Biosciences	1:50
CD63	AB_2573356	PE-Cy7	Rat	25-0631-82	NVG-2	eBioscience	1:200

References

1. Hoiseth, S. K. & Stocker, B. a. D. Aromatic-dependent Salmonella typhimurium are non-virulent and effective as live vaccines. *Nature* **291**, 238–239 (1981).
2. Kaniga, K., Bossio, J. C. & Galán, J. E. The Salmonella typhimurium invasion genes invF and invG encode homologues of the AraC and PulD family of proteins. *Molecular Microbiology* **13**, 555–568 (1994).
3. Hapfelmeier, S. *et al.* Role of the Salmonella pathogenicity island 1 effector proteins SipA, SopB, SopE, and SopE2 in Salmonella enterica subspecies 1 serovar Typhimurium colitis in streptomycin-pretreated mice. *Infect Immun* **72**, 795–809 (2004).
4. Di Martino, M. L., Ek, V., Hardt, W.-D., Eriksson, J. & Sellin, M. E. Barcoded Consortium Infections Resolve Cell Type-Dependent Salmonella enterica Serovar Typhimurium Entry Mechanisms. *mBio* **10**, e00603-19 (2019).
5. Mirold, S. *et al.* Salmonella Host Cell Invasion Emerged by Acquisition of a Mosaic of Separate Genetic Elements, IncludingSalmonella Pathogenicity Island 1 (SPI1), SPI5, and sopE2. *Journal of Bacteriology* **183**, 2348–2358 (2001).
6. Ehrbar, K., Friebel, A., Miller, S. I. & Hardt, W.-D. Role of the Salmonella Pathogenicity Island 1 (SPI-1) Protein InvB in Type III Secretion of SopE and SopE2, Two Salmonella Effector Proteins Encoded Outside of SPI-1. *Journal of Bacteriology* **185**, 6950–6967 (2003).
7. Porwollik, S. *et al.* Defined Single-Gene and Multi-Gene Deletion Mutant Collections in Salmonella enterica sv Typhimurium. *PLOS ONE* **9**, e99820 (2014).
8. Freed, N. E., Bumann, D. & Silander, O. K. Combining Shigella Tn-seq data with gold-standard E. coli gene deletion data suggests rare transitions between essential and non-essential gene functionality. *BMC Microbiology* **16**, 203 (2016).
9. Fahlgren, A., Avican, K., Westermark, L., Nordfelth, R. & Fällman, M. Colonization of Cecum Is Important for Development of Persistent Infection by Yersinia pseudotuberculosis. *Infection and Immunity* **82**, 3471–3482 (2014).
10. Drecktrah, D. *et al.* Dynamic Behavior of Salmonella-Induced Membrane Tubules in Epithelial Cells. *Traffic* **9**, 2117–2129 (2008).
11. Hapfelmeier, S. *et al.* The Salmonella Pathogenicity Island (SPI)-2 and SPI-1 Type III Secretion Systems Allow Salmonella Serovar typhimurium to Trigger Colitis via MyD88-Dependent and MyD88-Independent Mechanisms1. *The Journal of Immunology* **174**, 1675–1685 (2005).
