# Supplementary material for: A two-step activation mechanism enables mast cells to differentiate their response between extracellular and invasive enterobacterial infection
Source: Nat Commun. 2024 Jan 30;15:904. doi: 10.1038/s41467-024-45057-w (PMC10828507; doi:10.1038/s41467-024-45057-w)
Supplement: Supplementary file 3 — Reporting Summary [file 41467_2024_45057_MOESM3_ESM.pdf]

nature portfolio

Corresponding author(s): Gunnar Pejler, Mikael E. Sellin

Last updated by author(s): Dec 20, 2023

Reporting Summary

Nature Portfolio wishes to improve the reproducibility of the work that we publish. This form provides structure for consistency and transparency in reporting. For further information on Nature Portfolio policies, see our Editorial Policies and the Editorial Policy Checklist.

Statistics

For all statistical analyses, confirm that the following items are present in the figure legend, table legend, main text, or Methods section.

n/a	Confirmed	
☐	☒	The exact sample size (*n*) for each experimental group/condition, given as a discrete number and unit of measurement
☐	☒	A statement on whether measurements were taken from distinct samples or whether the same sample was measured repeatedly
☐	☒	The statistical test(s) used AND whether they are one- or two-sided<br>*Only common tests should be described solely by name; describe more complex techniques in the Methods section.*
☒	☐	A description of all covariates tested
☐	☒	A description of any assumptions or corrections, such as tests of normality and adjustment for multiple comparisons
☐	☒	A full description of the statistical parameters including central tendency (e.g. means) or other basic estimates (e.g. regression coefficient) AND variation (e.g. standard deviation) or associated estimates of uncertainty (e.g. confidence intervals)
☐	☒	For null hypothesis testing, the test statistic (e.g. *F*, *t*, *r*) with confidence intervals, effect sizes, degrees of freedom and *P* value noted<br>*Give P values as exact values whenever suitable.*
☒	☐	For Bayesian analysis, information on the choice of priors and Markov chain Monte Carlo settings
☒	☐	For hierarchical and complex designs, identification of the appropriate level for tests and full reporting of outcomes
☒	☐	Estimates of effect sizes (e.g. Cohen's *d*, Pearson's *r*), indicating how they were calculated

Our web collection on statistics for biologists contains articles on many of the points above.

Software and code

Policy information about availability of computer code

Data collection	No software was used for data collection.
Data analysis	RNA sequencing data was analyzed with R Studio 2022.12.0+3532 with R 4.2.2, using DESeq2 1.38. Graphpad Prism 10 was used for plotting and all other statistics analyses. For image analysis Fiji was used. FlowJo 10.8.1 was used for analyzing flow cytometry data.

For manuscripts utilizing custom algorithms or software that are central to the research but not yet described in published literature, software must be made available to editors and reviewers. We strongly encourage code deposition in a community repository (e.g. GitHub). See the Nature Portfolio guidelines for submitting code & software for further information.

Data

Policy information about availability of data

All manuscripts must include a data availability statement. This statement should provide the following information, where applicable:

- Accession codes, unique identifiers, or web links for publicly available datasets
- A description of any restrictions on data availability
- For clinical datasets or third party data, please ensure that the statement adheres to our policy

The RNA seq data generated in this study have been deposited in the GEO under accession number GSE223601. The rest of the data are available in the article, supplementary Information, or source data file. Source data are provided with this paper.

Research involving human participants, their data, or biological material

Policy information about studies with human participants or human data. See also policy information about sex, gender (identity/presentation), and sexual orientation and race, ethnicity and racism.

Reporting on sex and gender	NA
Reporting on race, ethnicity, or other socially relevant groupings	NA
Population characteristics	NA
Recruitment	NA
Ethics oversight	NA

Note that full information on the approval of the study protocol must also be provided in the manuscript.

Field-specific reporting

Please select the one below that is the best fit for your research. If you are not sure, read the appropriate sections before making your selection.

[x] Life sciences [ ] Behavioural & social sciences [ ] Ecological, evolutionary & environmental sciences

For a reference copy of the document with all sections, see nature.com/documents/nr-reporting-summary-flat.pdf

Life sciences study design

All studies must disclose on these points even when the disclosure is negative.

Sample size	Sample sizes were based on those used in previous and preliminary studies from our labs(s).
Data exclusions	No exclusions were made.
Replication	Experimental replicates are indicated in the figure legends, and each experiment was repeated at least two times.
Randomization	All experiments were conducted using randomly assigned animals, mast cell cultures derived from randomly assigned animals, or tissues derived from randomly assigned animals.
Blinding	Tissue slides used for manual counting were blinded to the experimenter, but distinction between uninfected and infected mice were in many cases still apparent due to differences in tissue architecture.

Reporting for specific materials, systems and methods

We require information from authors about some types of materials, experimental systems and methods used in many studies. Here, indicate whether each material, system or method listed is relevant to your study. If you are not sure if a list item applies to your research, read the appropriate section before selecting a response.

Materials & experimental systems

n/a	Involved in the study	
[ ]	[x]	Antibodies
[ ]	[x]	Eukaryotic cell lines
[x]	[ ]	Palaeontology and archaeology
[ ]	[x]	Animals and other organisms
[x]	[ ]	Clinical data
[x]	[ ]	Dual use research of concern
[x]	[ ]	Plants

Methods

n/a	Involved in the study	
[x]	[ ]	ChIP-seq
[ ]	[x]	Flow cytometry
[x]	[ ]	MRI-based neuroimaging

Antibodies

Antibodies used	Salmonella O Antiserum Factor 5 Rabbit BD-226601 Polyclonal Difco/MicLev 1:250<br>Akt AB_329827 Rabbit 9272 Polyclonal Cell Signaling 1:1000

Phospho-Akt (Ser473) AB_2315049 NA Rabbit 4060 D9E Cell Signaling 1:2000
CD16/32 AB_394657 Rat 553142 2.4G2 BD Biosciences 1:1000
TLR4 AB_469944 Alexa Fluor 488 Mouse 53-9041-82 UT41 Invitrogen/Thermo Fisher 1:100
Mouse IgG1 Isotype Control Alexa Fluor 488 Mouse MG120 Polyclonal Invitrogen/Thermo Fisher 1:100
CD45 AB_1645208 Alexa Flour 700 Rat 560510 30-F11 BD Biosciences 1:100
CD11b AB_468714 PE-Cy5 Rat 15-0112-82 M1/70 eBioscience/Thermo Fisher 1:100
CD11c AB_469590 PE-Cy7 Armenian hamster 25-0114-82 N418 eBioscience/Thermo Fisher 1:100
F4/80 AB_465923 PE Rat 12-4801-82 BMB Invitrogen/Thermo Fisher 1:100
CD45 AB_470499 Rat ab25386 I3/2.3 AbCam 1:50
CD18 AB_396701 Rat 557437 M18/2 BD Biosciences 1:50
CD63 AB_2573356 PE-Cy7 Rat 25-0631-82 NVG-2 eBioscience/Thermo Fisher 1:200
Goat-α-rabbit-IgG(H+L)-Cy3 (Molecular probes, #A10520, polyclonal, AB_2534029) 1:200
Goat-α-rabbit-IgG-HRP (1:10,000, Cell Signaling, #7074, polyclonal, AB_10697506, AB_11178535)
Goat-α-Rat-IgG(H+L)-AF647 (Invitrogen/Thermo Fisher, #1066650, AB_141778, polyclonal)

Validation

Primary antibodies used have been validated by the respective manufacturer, as specified in the product descriptions for the respective antibodies listed above, accessible through the homepage of each manufacturer; Difco/Miclev (www.miclev.se), Cell Signaling (www.cellsignal.com), BD Biosciences (www.bdbiosciences.com), Invitrogen/Thermo Fischer (www.thermofisher.com), eBioscience/Thermo Fisher (www.thermofisher.com), and Abcam (www.abcam.com).

Eukaryotic cell lines

Policy information about cell lines and Sex and Gender in Research

Cell line source(s)

LUVA cells were from Kerafast. Boner marrow-derived mast cells and peritoneal cell-derived mast cells were both from male and female mice.

Authentication

We bought LUVA cells from the vendor but did not authenticate them ourselves. Mast cells were routinely checked for CD117+ and FceRI+ surface expression by flow cytometry.

Mycoplasma contamination

The BMMC and PCMC cultures were not routinely tested for Mycoplasma contamination due to short-term culturing and repeated rederivation of fresh cultures with consistent results. Sentinel cell line cultures within the cell culture facility were tested for mycoplasma at regular intervals. LUVA cells used were likewise tested for Mycoplasma (negative), employing the Eurofins service (https://eurofinsgenomics.eu/en/genotyping-gene-expression/applied-genomics-services/mycoplasmacheck/).

Commonly misidentified lines
(See ICLAC register)

We did not use commonly misidentified cell lines.

Animals and other research organisms

Policy information about studies involving animals; ARRIVE guidelines recommended for reporting animal research, and Sex and Gender in Research

Laboratory animals

For BMMCs and PCMCs: Mus musculus C57BL/6 from own breeding, ages 8-14 weeks were used. For experiments involving Tlr4-/-BMMCs, B6(Cg)-Tlr4tm1.2Karp/J (#029015) and corresponding C57BL/6J WT (#000664), 8 weeks old mice were purchased from The Jackson Laboratory. Animal tissues were processed immediately after arrival. In vivo experiments were performed with 8-weeks old CBA mice from Charles River. Housing was conducted in dedicated facilities (Umeå University, Umeå, and SVA, Uppsala, respectively) using individually ventilated cages kept under ambient temperature and diurnally alternating light-dark-cycling (12h:12h).

Wild animals

No wild animals were used in this study.

Reporting on sex

We did not observe differences between male and female origin among mouse-derived mast cells. The in vivo experiments were performed on female mice.

Field-collected samples

No field-collected samples were used in this study.

Ethics oversight

For generation of mast cell cultures, all experimental procedures were approved by the local animal ethics committee of Uppsala (Dnr. 5.8.18-05357/2018). In vivo experiments were approved by the local animal ethics committee of Umeå (Dnr. A27-17).

Note that full information on the approval of the study protocol must also be provided in the manuscript.

Plants

Seed stocks	NA
Novel plant genotypes	NA
Authentication	NA

Flow Cytometry

Plots

Confirm that:

- [x] The axis labels state the marker and fluorochrome used (e.g. CD4-FITC).
- [ ] The axis scales are clearly visible. Include numbers along axes only for bottom left plot of group (a 'group' is an analysis of identical markers).
- [x] All plots are contour plots with outliers or pseudocolor plots.
- [ ] A numerical value for number of cells or percentage (with statistics) is provided.

Methodology

Sample preparation	Mast cells and bone marrow cells were prepared as described in the methods section of the manuscript.
Instrument	MACSQuant VYB (Miltenyi Biotec)
Software	For acquisition: MACS Quantify Software 2.11 (Miltenyi Biotec)<br>For analysis: FlowJo 10.8.1 (BD Biosciences)
Cell population abundance	At least 10,000 events were acquired. For BMMC and PCMC cultures, the cell population purity was >>95%.
Gating strategy	Cells were gated with FSC-H and FSC-A for single cells, and based on FSC-A and SSC-A, cells were distinguished from debris. For all infection experiments, gating was based on two clearly distinguished population and comparison to uninfected cells. For TLR4 staining, TLR4+ cells were distinguished by isotype staining. For the immune cell identification in bone marrow, 10 million live bone marrow cells were used for comparison. For gating examples, see Figure S8.

- [x] Tick this box to confirm that a figure exemplifying the gating strategy is provided in the Supplementary Information.
