# Supplementary figures and images for: A two-step activation mechanism enables mast cells to differentiate their response between extracellular and invasive enterobacterial infection
Source: Nat Commun. 2024 Jan 30;15:904. doi: 10.1038/s41467-024-45057-w (PMC10828507; doi:10.1038/s41467-024-45057-w)

## Immunoblot raw images

Figure 5c

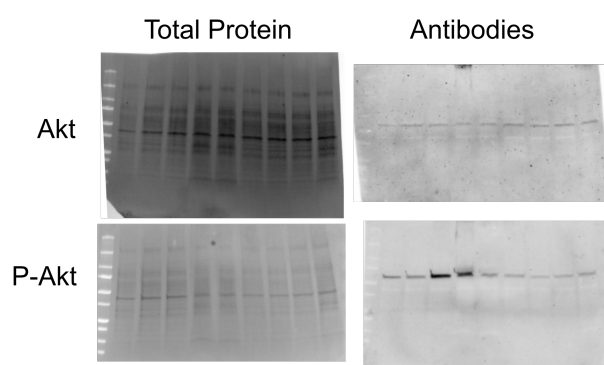

Figure 5d

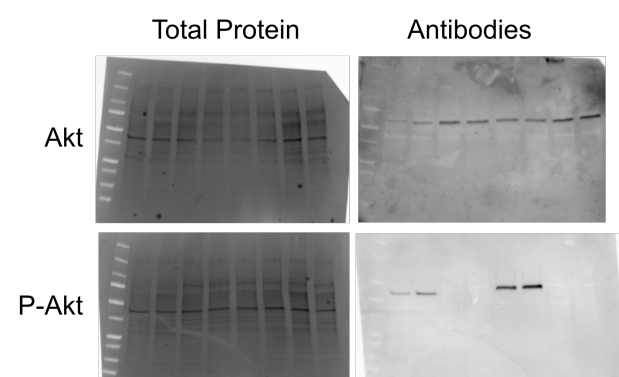

Figure S6e

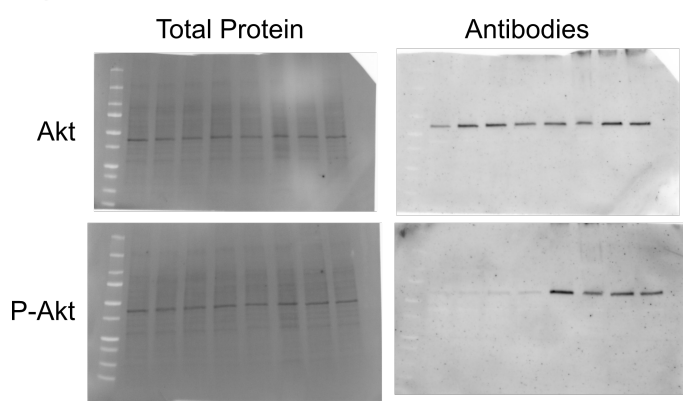

Supplement: Supplementary file 4 — Source Data [file 41467_2024_45057_MOESM4_ESM.zip › Source data_Immunoblots.pdf]
